# Longitudinal study of the *mcr-1* gene prevalence in Spanish food-producing pigs from 1998 to 2021 and its relationship with the use of polymyxins

**DOI:** 10.1186/s40813-022-00255-0

**Published:** 2022-03-17

**Authors:** Pedro Miguela-Villoldo, Miguel A. Moreno, David Rodríguez-Lázaro, Alejandro Gallardo, Marta Hernández, Tania Serrano, José L. Sáez, Cristina de Frutos, Montserrat Agüero, Alberto Quesada, Lucas Domínguez, María Ugarte-Ruiz

**Affiliations:** 1 VISAVET Health Surveillance Centre, Universidad Complutense de Madrid, Avenida Puerta de Hierro, s/n, 28040 Madrid, Spain; 2 Departamento de Sanidad Animal, Facultad de Veterinaria, Universidad Complutense de Madrid, Avenida Puerta de Hierro, s/n, 28040 Madrid, Spain; 3 Área de Microbiología, Departamento de Biotecnología y Ciencia de los Alimentos, Universidad de Burgos, Burgos, Spain; 4 Departamento de Bioquímica, Biología Molecular y Genética, Facultad de Veterinaria, Universidad de Extremadura, Avenida de la Universidad s/n, 10003 Cáceres, Spain; 5 Laboratorio de Biología Molecular y Microbiología, Instituto Tecnológico Agrario de Castilla y León, Valladolid, Spain; 6 Tecnologías y Servicios Agrarios S.A, Madrid, Spain; 7 Subdirección General de Sanidad e Higiene Animal y Trazabilidad, Ministerio de Agricultura y Pesca, Alimentación y Medio Ambiente, Madrid, Spain; 8 Laboratorio Central de Veterinaria, Algete, Madrid, Spain; 9 INBIO G+C, Universidad de Extremadura, Avenida de la Universidad s/n, 10003 Cáceres, Spain

**Keywords:** Colistin, Antimicrobial resistance, Real-time PCR, Swine, Resistance genes

## Abstract

**Background:**

Resistance to colistin was an uncommon phenomenon traditionally linked to chromosome point mutations, but since the first description of a plasmid-mediated colistin-resistance in late 2015, transmissible resistance to colistin has become a Public Health concern. Despite colistin is considered as a human last resort antibiotic, it has been commonly used in swine industry to treat post-weaning diarrhoea in piglets. However, the progressively increase of colistin resistance during the last decade led to the Spanish Medicines and Healthcare Products Agency (AEMPS) to launch a strategic and voluntary plan aimed to reduce colistin consumption in pig production. Our longitudinal study (1998–2021) aimed to evaluate the trend of colistin resistance mediated through the *mcr-1* mobile gene in Spanish food-producing pig population and compare it with published polymyxin sales data in veterinary medicine to assess their possible relationships.

**Results:**

The first *mcr-1* positive sample was observed in 2004, as all samples from 1998 and 2002 were *mcr-1* PCR-negative. We observed a progressive increase of positive samples from 2004 to 2015, when *mcr-1* detection reached its maximum peak (33/50; 66%). From 2017 (27/50; 54%) to 2021 (14/81; 17%) the trend became downward, reaching percentages significantly lower than the 2015 peak (*p* < 0.001). The abundance of *mcr-1* gene in PCR-positive samples showed a similar trend reaching the highest levels in 2015 (median: 6.6 × 10^4^ *mcr-1* copies/mg of faeces), but decreased significantly from 2017 to 2019 (median 2.7 × 10^4^, 1.2 × 10^3^, 4.6 × 10^2^ *mcr-1* copies/mg of faeces for 2017, 2018 and 2019, respectively), and stabilizing in 2021 (1.6 × 10^2^ *mcr-1* copies/mg of faeces) with similar values than 2019.

**Conclusions:**

Our study showed the decreasing trend of colistin resistance associated to *mcr-1* gene, after a previous increase from among 2004–2015, since the European Medicines Agency and AEMPS strategies were applied in 2016 to reduce colistin use in animals, suggesting a connection between polymyxin use and colistin resistance. Thus, these plans could have been effective in *mcr-1* reduction, reaching lower levels than those detected in samples collected 17 years ago, when resistance to colistin was not yet a major concern.

## Background

Resistance to colistin, antibiotic belonging to polymyxins family, was traditionally linked to chromosome point mutations, but since the first description of a plasmid-mediated colistin-resistance mechanism mediated by a family of *mcr* genes on late 2015 [1], transmissible resistance to colistin has become a Public Health concern. Although up to ten members of this family have been described (*mcr-1* to *mcr-10*) [2–10], *mcr-1* is the most extended and frequently detected in many countries around the world [10–12], demonstrating it as an excellent indicator for monitoring colistin resistance [13].

The increase of human infections due to multidrug-resistant (MDR) Gram-negative bacteria, especially those producing extended-spectrum beta-lactamases (ESBL) and carbapenemases, coupled with the lack of the development of novel antimicrobials, led to the reintroduction of colistin by the mid-1990s to treat these human infections, as it was often the only effective antimicrobial against them [14]. Therefore, 3rd- and 4th-generation cephalosporins along with colistin, are considered as critically important for both human and animal health [15, 16] and they are recently classified into the “RESERVE” group in the World Health Organisation (WHO) AWaRe classification [17]. Nonetheless, colistin was extensively used to treat post-weaning diarrhoea (PWD) in piglets [18].

Thus, following the European Medicines Agency’s (EMA) advice about the use of colistin in animals, the Spanish Medicines and Healthcare Products Agency (AEMPS) launched in 2016 the “*Reduce Porcino*” plan, a strategic and voluntary plan aimed to reduce the colistin consumption to 5 mg/PC in three years in pig production and control the possible alternative use of neomycin and apramycin. This initiative was followed by the 80% of Spanish pig producers contributing to a decrease in colistin consumption of 97% (AEMPS, 12/2019) [19].

A potential relationship between colistin resistance and polymyxins use has been pointed out [20–22], which is consistent with the findings observed in our previous studies [23], so the screening of levels of colistin-resistant bacteria has become an important procedure to assess the effectiveness of the reduction of antimicrobial use over colistin resistance. However, there is a lack of studies that jointly analyse the evolution of both colistin resistance and polymyxins use. So, we aimed to monitoring resistance to colistin mediated by the mobile *mcr-1* gene in the Spanish food-producing pig population and compare it with published data of polymyxins sales in veterinary medicine to assess their relationships.

## Results

### Detection of *mcr-1* in pig samples

**Fig. 1:**
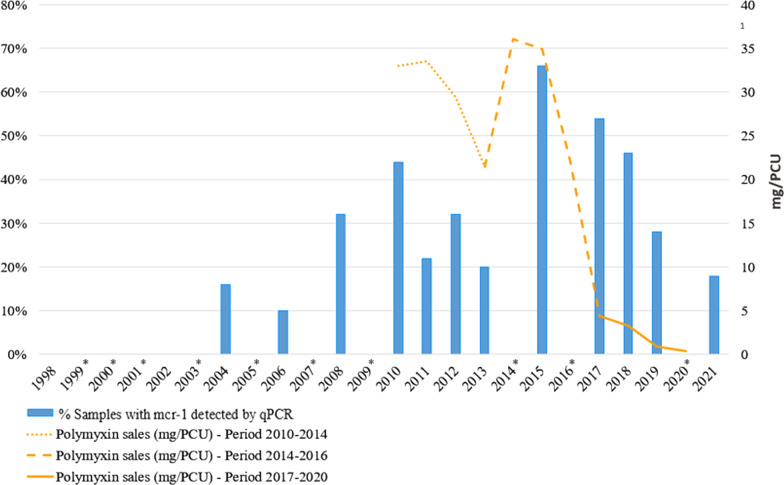
Evolution of the percentage of pig samples positive to *mcr-1* gene and the polymyxin sales reported in Spain for veterinary medicine. Data on polymyxin sales were available from 2010 to 2020 (the latest year reported currently by ESVAC). These data was represented in three different periods with the same data collection system, as it was specified in the ESVAC report for spain. PCU: population correction unit. *No *mcr-1* gene prevalence dta in the years 1999, 2000, 2001, 2003, 2005, 2007, 2009, 2014, 2016 and 2020

The first *mcr-1* PCR-positive sample was observed in 2004 sampling, as all samples from 1998 and 2002 were *mcr-1* PCR-negative (Fig. 1). The percentage of positive samples increased from 2004 (8/50; 16%, 95% CI = 7.2% to 29.1%) until 2010 (22/50; 44%, 95% CI = 30.0% to 58.8%), showing a statistically significant difference (*p* = 0.002) between both years. From 2010 to 2013, it was noticed an irregular progression characterized by a slight decrease in the number of positive samples (no statistically significant), followed by a significant increase in 2015 (*p* = 0.027) compared to data from 2010. So, we observed an increase of positive samples from 2004 until 2015, when *mcr-1* PCR positive-samples reached its maximum value (33/50; 66%, 95% CI = 51.2% to 78.8%). Finally, from 2017 (27/50; 54%, 95% CI = 39.3% to 68.2%) to 2021 (9/50; 18%, 95% CI = 8.6% to 31.4%) the *mcr-1* PCR positive samples decreased, reaching percentages significantly lower than the 2015 peak (*p* < 0.001) and similar to years prior to 2008 (16/50; 32%, 95% CI = 19.5% to 46.7%) (Fig. 1).

These data were compared with the Spanish polymyxin sales data published in the Eleventh European Surveillance of Veterinary Antimicrobial Consumption (ESVAC) Report [24] and a declining progression of both data series, especially since 2015, was noticed. Although the polymyxin sales showed a steeper drop than the *mcr-1* positive samples percentages, both decreasing trends suggest that the reduction of polymyxins sales could have had a strong influence on the decrease of *mcr-1* positive samples, among other factors (Fig. 1).

### Abundance of *mcr-1* in pig samples

**Fig. 2:**
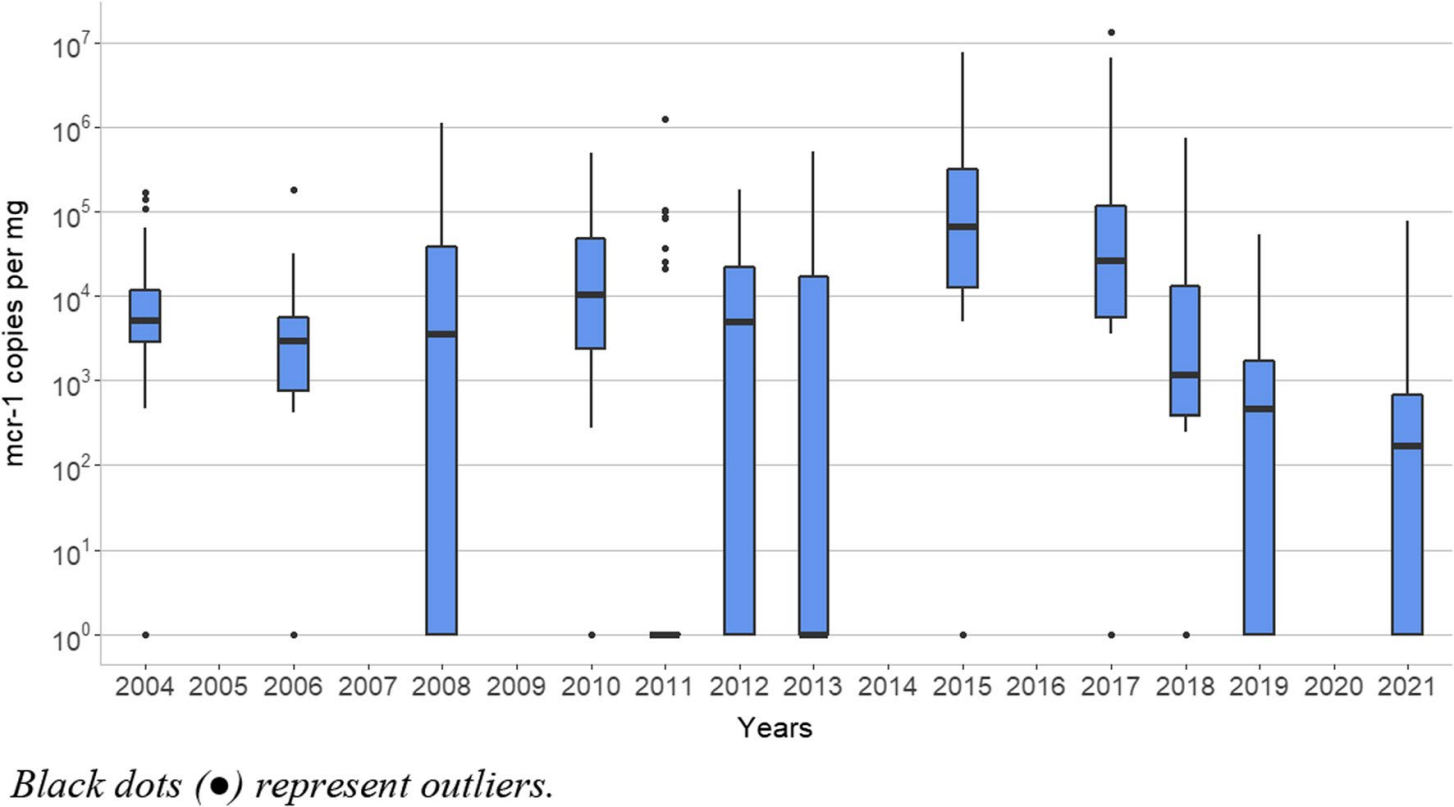
Box-plot diagram of the yearly distribution of mcr-1 gene abundance (expressed in copies/mg faecal content) in Spanish food-producing pigs from positives caecal samples. Black dots represent outliers

The abundance (copies/mg caecal content) of the *mcr-1* gene in those years with PCR-positive samples showed a similar trend to the percentage of *mcr-1* gene positive samples (Fig. 2). The median values from 2015 and 2017 were similar (6.6 × 10^4^ and 2.7 × 10^4^ *mcr-1* copies/mg, respectively), but the median of *mcr-1* gene quantification remarkably dropped in 2018 (1.2 × 10^3^ *mcr-1* copies/mg; *p* < 0.01). This decrease continued in 2019 (median 4.6 × 10^2^ *mcr-1* copies/mg) and it was stabilised in 2021 (median 1.6 × 10^2^ *mcr-1* copies/mg). Thus, both indicators, the number of *mcr-1* positive samples and the amount of *mcr-1* copies, showed a similar decrease trend since 2015.

## Discussion

Colistin resistance mediated by *mcr* mobile genes is a great concern around the world [25]. There are some studies reporting changes in the *mcr* abundance by culture and molecular analysis of bacterial isolates [26, 27], but only few studies have focused on samples rather than strains, such as livestock manure [28], human and pets faeces [29, 30] or faeces from food-producing animals [29]. Similarly, few studies have assessed the evolution of the presence of *mcr* genes in a given animal, food or human related environment along a large period of time. Consequently, the results obtained in our study may provide a new perspective on the spread of the *mcr-1* gene in the highly relevant bacterial context represented by the intestinal microbiota. However, since the effect of prolonged storage of fecal samples on the integrity of their DNA, which could be affected by chemical or enzymatic degradative processes, is unknown, its representativeness should be considered with caution, especially in the early stages of this study that reaches up to 23 years old.

We also compared the evolution of *mcr-1* presence in faecal samples with polymyxin use in animal production, which makes this work useful to assess the current decisions about colistin restrictions recommended in the European Union (EU). However, the Spanish antimicrobial sales data collection system detected under-reporting for the period 2010 – 2013, so the data for these years are underestimates. In addition, datasets included veterinary medicinal products (VMPs) declared by marketing authorisation holders (MAHs) which were replaced by retailers as sales data providers for the period 2017–2020. Due to these differences, it is difficult to assess the trends for the periods 2010–2014, 2014–2016 and 2017–2020, and comparisons between them should be made with caution [31]. Thus, these periods were assessed separately.

In accordance with the aim of the AEMPS colistin reduction programmes launched to decrease the use of colistin in different food-producing animal species such as swine [32], polymyxin sales in Spain dropped down from 22.02 mg/PCU in 2016 to 4.4 mg/PCU in 2017, 3.3 mg/PCU in 2018, 0.9 mg/PCU in 2019 and 0.4 mg/PCU in 2020, also achieving the EMA’s main objective of reducing the antibiotic consume under 5 mg/PCU in a period of 3 to 4 years from 2016 [24, 33].

Interestingly, the first detection of *mcr-1* in pigs in Spain (2004) was coincidental with the suppression of zinc oxide (ZnO) as an additive food complement for feeding piglets during 2003–2004. ZnO has been used traditionally as a food additive in piglets; however, there was an increasing concern regarding the potential risk for the environment derived from its use. Thus, the Committee for Medicinal Products for Veterinary Use (CVMP) of EMA considered that the benefits of using ZnO did not exceed the risks to the environment and consequently, this substance was banned in 2003 until the 15th November 2004, when ZnO was officially registered as a premix [34]. In addition, the Scientific Committee on Animal Nutrition (SCAN) of EU recommended, also in 2003, a reduction of ZnO levels in feeding-stuffs as they were higher than necessary to supply the physiological requirements of the animals [35]. During the ban, colistin emerged as an alternative to treat PWD in piglets and ZnO was mostly replaced by colistin. Interestingly, in the 2004–2010 period we observe an increase in the number of *mcr-1*-carrying samples which could be related with the widening use of this antibiotic.

The drop of *mcr-1* positive samples for the period 2011–2013 compared with 2010 was coincident with a reduction in the piglets’ production in Spain, as it is represented in Fig. 1; however, the decrease of polymyxin sales could be more related with the changes in the antimicrobial sales data collection method above mentioned. In 2015, an increase of *mcr-1* positive samples was observed with the highest proportion of samples of our study. According to the antimicrobial sales data compiled by ESVAC, Spain had in 2015 the highest figures of polymyxins sales in the EU (34.9 mg/PCU) [33]; however, after the Spanish voluntary strategic plan launched by the AEMPS, polymyxin sales remarkably decrease in the next years (4.4, 3.3, 0.9 and 0.4 mg/PCU from 2017, 2018, 2019 and 2020 respectively) [24].

From 2017 to 2021, the percentage of *mcr-1-*positive samples decreased in agreement with the reduction in the polymyxin sales, reaching levels of colistin resistance lower than those obtained in 2008. This phenomenon has been observed in others UE countries, such as Italy [26], Germany [36] among others, where implemented strategies to limit the use of colistin also produced a parallel decrease between colistin resistance and antibiotic consume (data expressed in mg/kg of estimated biomass) in animals, especially pigs and poultry showing a statistically significant association [22]. Similar results has been also reported in China [20].

Our retrospective longitudinal study suggest that colistin resistance associated to *mcr-1* gene is related to polymyxins sales, as different studies [20, 26, 37] and authorities [22, 32, 38] have also pointed out. In this context, we observed a decrease in both colistin resistance and polymyxins sales in the 2017 to 2021 period, and the proportion of positive samples and the abundance of *mcr-1* gene in positive samples reached levels lower than those obtained even in 2004. This context reinforces the food-producing industry against future changes in animal care legislations, such as the next ZnO in the EU ban planned for 2022 which was approved in 2017 [39] and for which pig industry will be better prepared than 2003, when ZnO was mostly replaced by colistin and led us to the current situation.

Colistin resistance due to *mcr-1* gene showed a slower decrease than colistin sales probably due to the genetic environment where *mcr-1* gene was encoded. The genome location of *mcr-1* gene may play a determinant role in its dispersion and abundance [40–42]. The *mcr-1* gene is usually encoded in plasmids, allowing the horizontal gene transference and thus a faster spread of the resistance gene to other bacteria which become resistant to colistin [1, 37]. Hypothetically, reduced use of colistin would cause a drop in the selective pressure on the intestinal bacterial population. This would allow colistin susceptible bacteria to survive and outcompete colistin resistant bacteria, due to the fitness cost associated with maintaining plasmid-encoded genes [42, 43]. This hypothesis seems to be in concordance with data from the joint report by European Centre for Disease Prevention and Control (ECDC), European Food Safety Authority (EFSA) and EMA (Jiacra III report) [22]. However, there are evidences of the co-selection of *mcr* genes in a free colistin selective pressure environment in plasmid containing *mcr* genes and resistance genes against other antimicrobial families such as ESBL (*bla*_*CTX-M*_ and *bla*_*SHV*_*)*, *cml* or *sul* (chloramphenicol and sulfamethoxazole resistance genes respectively) [44–47]. Another less frequent factor that could allow the persistence of colistin resistance in the host strain is the presence of *mcr-1* gene in the bacterium chromosome, usually flanked by insert sequences [40, 48]. Thus, the location of colistin resistance genes may be determinant in the fight against colistin resistance, which is an open window for further studies.

## Conclusions

Our results showed a connection between polymyxin sales and colistin resistance in Spanish pigs for food-production in a range of nine years, and a decreasing trend of colistin resistance (*mcr-1* gene) since the EMA and AEMPS strategies were applied in 2016 to reduce colistin use in animals. Thus, our data suggest to the reduction plans would have had an important effect in the fight against colistin resistance (*mcr-1* gene), since its presence in pigs have returned to slightly lower levels than those detected in samples collected 17 years ago, when resistance to colistin was not yet a major concern.

## Methods

### Samples

The collection of caecal samples from healthy pigs taken at slaughterhouse level under the National antimicrobial resistance surveillance program (1998 to 2021), driven by the Spanish Ministry of Agriculture, Fisheries and Food (except 2018) were used for this study. Sampling from 2018 was covered by the VISAVET Health Surveillance Centre, under the same conditions as the National antimicrobial surveillance programs to maintain the homogeneity of the sampling.

We have combined a retrospective study, based on frozen (ten-fold diluted in sterile water and glycerol prior to freezing at − 40 °C) samples collected from 1998 to 2017, with a prospective study done with fresh samples taken in 2018, 2019 and 2021.

We randomly selected 50 samples per year (1998, 2002, 2004, 2006, 2008, 2010, 2011, 2012, 2013, 2015, 2017, 2018, 2019 and 2021) accomplishing a total number of 700 samples.

### Quantitative assay for *mcr-1*

Before processing, frozen samples were thawed at room temperature. From this step, all samples were processed in the same way. Direct DNA extraction was carried out using a commercial kit (FASTI001-1 FavorPrep Stool DNA Isolation Mini Kit, Favorgen-Europe, Vienna) following the manufacturer’s specifications (elution volume of 200 µL), coupled to a specific SYBRGreen real-time PCR assay for quantitative detection of the *mcr-1* gene (qPCR), described previously [49] and further validated in our lab [13]. Samples were considered positive if their quantitative DNA values were > 1.00 × 10^2^ fg/µL, equivalent to 1.58 × 10^3^ *mcr-1* copies/mg caecal content.

### Statistical analysis

The data obtained by qPCR was analysed using a T-test after normalisation by logarithmic transformation into Log_10_, and Chi-square test was used to compare the frequencies of *mcr-1* positive samples observed each year. A difference was considered significant when the *p* was < 0.05.

## Data Availability

All datasets used in this study are available from the corresponding author on reasonable request.

## Abbreviations

AEMPS: Spanish Medicines and Healthcare Products Agency
CI: Confidence Interval
ECDC: European Centre for Disease Prevention and Control
EFSA: European Food Safety Authority
EMA: European Medicines Agency
ESBL: Extended-spectrum beta-lactamases
ESVAC: European Surveillance of Veterinary Antimicrobial Consumption
EU: European Union
MAHs: Marketing Authorisation Holders
MDR: Multidrug-resistant
PCR: Polymerase Chain Reaction
PCU: Population Correction Unit
PWD: Post-weaning diarrhoea
SCAN: Scientific Committee on Animal Nutrition
VMPs: Veterinary Medicinal Products
WHO: World Health Organisation
ZnO: Zinc oxide
